# Thank you to the *Journal of the Medical Library Association* reviewers in 2017

**DOI:** 10.5195/jmla.2018.425

**Published:** 2018-04-01

**Authors:** Katherine G. Akers

**Affiliations:** Editor-in-Chief, *Journal of the Medical Library Association*

## Abstract

The *Journal of the Medical Library Association (JMLA)* sincerely thanks our peer reviewers in 2017.

The *Journal of the Medical Library Association (JMLA)* sincerely thanks the 154 reviewers in 2017 who helped vet and improve the quality of work published in our journal.

The *JMLA* is always looking to expand our pool of reviewers who can critically comment on any topic of research or practice in health sciences librarianship. If you are interested in serving as a peer reviewer for the *JMLA,* please send your CV to the editor-in-chief at jmla@journals.pitt.edu.

## 2017 *JOURNAL OF THE MEDICAL LIBRARY ASSOCIATION* REVIEWERS

Nancy Adams

Nancy J. Allee, AHIP

Kristine M. Alpi, AHIP

Katelyn Angell

Kenning Arlitsch

Nell Aronoff

Caitlin J. Bakker, AHIP

Michael Eliot Bales

Becky Baltich Nelson

Jill Barr-Walker

Robert D. Beckett

Joeran Beel

Christopher Belter

Catherine Boden

Jill T. Boruff, AHIP

Paul Bracke

Wichor Bramer

Marci Brandenburg

Heather Brown

Nicole Capdarest-Arest, AHIP

Alexander James Carroll

Thane Chambers

Deborah H. Charbonneau

Yibu Chen

Nicole Contaxis

Marisa Conte

I. Diane Cooper, AHIP

Joseph Costello

Jill Crawley-Low

Andrew Creamer

Vicki F. Croft, FMLA, AHIP

Prudence Dalrymple, AHIP

Kate Daniels, AHIP

Ariel Deardorff

Antonio P. DeRosa, AHIP

Robin Desmeules

Jo Dorsch, AHIP, FMLA

Kathel Dunn

Martha Earl, AHIP

Erin RB Eldermire

Jonathan Eldredge, AHIP

Keith D. Engwall, AHIP

Julia M. Esparza, AHIP

Alison Farrell

Lisa Federer, AHIP

Barbara Folb

Erin Foster

Margaret Jane Foster, AHIP

Rick Fought, AHIP

Suzanne Fricke, AHIP

Julie Glanville

Abigail Goben

Sally Gore

Kelsey Grabeel, AHIP

Adelia Grabowsky

Karen Elizabeth Gutzman

Laura J. Hall

Margaret Henderson, AHIP

Jennifer Herron

Toni Hoberecht, AHIP

Kristi L. Holmes

Carol L. Howe

Ella Hu

Jeffrey Huber

Shanda L. Hunt

Emily Margaret Johnson, AHIP

Rebecca McKay Johnson, AHIP

Timothy P. Johnson

Dixie A. Jones, AHIP

Douglas Joubert

Kellie Kaneshiro, AHIP

Jill R. Kavanaugh, AHIP

Erin Kerby

Alla Keselman

Andrea M. Ketchum, AHIP

Sujin Kim

Daniel Kipnis

Stephen Kiyoi, AHIP

Molly Knapp, AHIP

Amy Knehans, AHIP

Laura Koppen

Petros Kostagiolas

Fred Willie Zametkin LaPolla

Janna Lawrence, AHIP

Brenda M. Linares, AHIP

Anne M. Linton, AHIP

Ayaba Logan

Diana Nelson Louden

Ya-Ling Lu

Mark MacEachern

Keith C. Mages, AHIP

Shaheen Majid

Lisa Mastin

Sarah McClung

Karen McElfresh, AHIP

Misa Mi, AHIP

Elizabeth Moreton

Martin Morris

Beverly Murphy, AHIP, FMLA

Bethany Myers, AHIP

Joey Nicholson

Tyler Nix

Rob O’Reilly

Jessica R. Page, AHIP

Robin M. N. Parker

Carol L. Perryman

Shenita Peterson

Jodi L. Philbrick, AHIP

Elizabeth Pienaar

JJ Pionke

T. Scott Plutchak, AHIP, FMLA

Kimberly Powell

Zahra Premji

Neil H. Rambo

Rebecca Raszewski, AHIP

Rebecca Raworth

Kevin Read

Sophie M. Regalado, AHIP

David Resnik

Melissa L. Rethlefsen, AHIP

Rebecca Reznik-Zellen

Stephanie Roth, AHIP

Sarah Safranek

Alexandra Sarkozy

Cathy Sarli, AHIP

Lou Ann Scarton

Cynthia Schmidt

Carolyn Schubert

Stephanie J. Schulte

Jean Shipman, AHIP, FMLA

Jean Song

Ansley Stuart, AHIP

Alisa Surkis

Stephanie Swanberg, AHIP

Natalie Tagge

Maria Tan

Nancy Tannery

Marilyn Teolis, AHIP

Nicole Theis-Mahon

Lorraine Toews

Efren Torres Jr.

Whitney Ann Townsend

Vedana Vaidhyanathan

Emily Vardell

Sarah May Visintini

Rachel R. Walden

Verma Walker

Amanda Wanner, AHIP

Erin Watson, AHIP

Monique Wessels

Julia Charma Whelan

Jeff D. Williams, AHIP

Kristen L. Young, AHIP

Kathy Zeblisky, AHIP

